# Dissection of Molecular Processes and Genetic Architecture Underlying Iron and Zinc Homeostasis for Biofortification: From Model Plants to Common Wheat

**DOI:** 10.3390/ijms21239280

**Published:** 2020-12-05

**Authors:** Jingyang Tong, Mengjing Sun, Yue Wang, Yong Zhang, Awais Rasheed, Ming Li, Xianchun Xia, Zhonghu He, Yuanfeng Hao

**Affiliations:** 1 Institute of Crop Sciences, Chinese Academy of Agricultural Sciences (CAAS), 12 Zhongguancun South Street, Beijing 100081, China; tongjingyang02@163.com (J.T.); smj970626@163.com (M.S.); wanyue_luna@163.com (Y.W.); zhangyong05@caas.cn (Y.Z.); liming08@caas.cn (M.L.); xiaxianchun@caas.cn (X.X.); 2 International Maize and Wheat Improvement Center (CIMMYT) China Office, c/o CAAS, 12 Zhongguancun South Street, Beijing 100081, China; arasheed@qau.edu.pk; 3 Department of Plant Sciences, Quaid-i-Azam University, Islamabad 45320, Pakistan

**Keywords:** iron, micronutrient, orthologous genes, wheat, zinc

## Abstract

The micronutrients iron (Fe) and zinc (Zn) are not only essential for plant survival and proliferation but are crucial for human health. Increasing Fe and Zn levels in edible parts of plants, known as biofortification, is seen a sustainable approach to alleviate micronutrient deficiency in humans. Wheat, as one of the leading staple foods worldwide, is recognized as a prioritized choice for Fe and Zn biofortification. However, to date, limited molecular and physiological mechanisms have been elucidated for Fe and Zn homeostasis in wheat. The expanding molecular understanding of Fe and Zn homeostasis in model plants is providing invaluable resources to biofortify wheat. Recent advancements in NGS (next generation sequencing) technologies coupled with improved wheat genome assembly and high-throughput genotyping platforms have initiated a revolution in resources and approaches for wheat genetic investigations and breeding. Here, we summarize molecular processes and genes involved in Fe and Zn homeostasis in the model plants *Arabidopsis* and rice, identify their orthologs in the wheat genome, and relate them to known wheat Fe/Zn QTL (quantitative trait locus/loci) based on physical positions. The current study provides the first inventory of the genes regulating grain Fe and Zn homeostasis in wheat, which will benefit gene discovery and breeding, and thereby accelerate the release of Fe- and Zn-enriched wheats.

## 1. Introduction

A multitude of trace metals, such as iron (Fe), zinc (Zn), copper (Cu), and manganese (Mn), are essential micronutrients for both plant growth and human health due to their pivotal roles in key metabolic reactions and myriad biochemical functions [1,2]. Among these minerals, Fe and Zn are thought to have vital biological relevance and clinical significance for global public health [3]. In plants, Fe is responsible for regulating photosynthesis, mitochondrial respiration, and protein stability, and is one of the cofactors in electron or oxygen transfer [1,4,5]. Zn is a key cofactor for protein function (e.g., zinc finger proteins), and is required for numerous plant physiological processes such as photosynthesis, signal transduction, and maintenance of CO_2_ levels in mesophyll [1,6,7]. With Fe and Zn deficiency, plants frequently show chlorosis and reduced photosynthesis, resulting in significantly reduced yield [8,9]. Millions of people worldwide suffer from micronutrient malnutrition, with, most prevalently, approximately two billion people with Fe and/or Zn deficiencies [10]. About 40% of children and pregnant women globally tend to be impacted by iron-deficiency anemia, which affects cognitive development and decreases immunity function [11,12]. Zn deficiency results in stunted growth and mental retardation in children worldwide [13]. Among the three primary interventions to surmount micronutrient malnutrition, biofortification, which increases the intrinsic nutritional content in edible crop plants, is thought to be the most sustainable method [14]. 

Wheat (*Triticum aestivum* L.) is one of the most widely grown and consumed crops, and is therefore recognized as an appropriate target for biofortification [15]. However, most common wheat cultivars have suboptimal concentrations of Fe and Zn [16]. Side-by-side comparisons of high- and low-yielding cultivars of crops in the past few decades have provided strong evidence that there can be trade-offs between the yield and content of some nutrients caused by breeding objectives highly focused on yield rather than other traits, such as micronutrient content [17,18,19,20,21]. Hence, it is imperative for scientists to re-introduce previously neglected current wheat breeding gene pools and increase grain Fe/Zn to an appropriate level. To achieve this, it is essential to identify as many as Fe/Zn-related QTL (quantitative trait locus/loci) or genes for wheat biofortification.

Although limited molecular progress has been made to understand Fe and Zn regulatory networks in wheat, substantial numbers of genes involved in Fe and Zn homeostasis in model plants such as the non-graminaceous plant *Arabidopsis thaliana* and graminaceous plant rice (*Oryza sativa* L.) have been extensively studied [22,23]. Comparative genomics approaches have facilitated the identification of putative orthologous genes as potential candidates for wheat Fe and Zn homeostasis [24,25]. This approach became particularly attractive when the fully annotated wheat reference genome of Chinese Spring RefSeq v.1.0 was released [26]. Concurrent with rapidly evolving high-throughput and cost-effective genotyping systems (e.g., array-based and sequencing-based platforms), a large number of Fe and Zn loci have been excavated through QTL mapping and association analysis [27,28]. Moreover, high-throughput phenotyping technologies, such as inductively coupled plasma–mass spectrometry (ICP-MS), X-ray fluorescence spectrometry, and newly emerged synchrotron-based X-ray fluorescence microscopy, are also making rapid advances, complementing deep genetic analysis and increasing the efficiency of Fe/Zn gene discovery in hexaploid wheat [29,30].

In this review, we will firstly recapitulate extant molecular processes in Fe and Zn homeostasis in the model plants *Arabidopsis* and rice, and summarize the genes implicated in Fe and Zn modulation. Secondly, sequences of the above genes were retrieved in BLAST analysis to obtain orthologous genes in wheat; these genes and previously located QTL for wheat Fe and Zn metabolism were jointly assembled on wheat chromosomes and stable genetic loci were identified based on QTL-rich clusters (QRCs). Thirdly, different strategies for wheat Fe and Zn biofortification are proposed by leveraging genetic basis. 

## 2. Molecular Mechanisms Regulating Fe and Zn Homeostasis in Model Plants 

Despite high abundance in the crust of the earth, Fe is less accessible to plants as it primarily exists as insoluble ferric oxides, especially in aerobic conditions at neutral or alkaline pH [31]. In response to the limited Fe availability in soil and potential Fe deficiency, plants have developed delicate mechanisms to tightly regulate Fe homeostasis, including Fe acquisition, translocation, and storage. Zn is also a crucial element and serves as a cofactor for over 1200 proteins in plants [32]. Zn absorption is a complex physiological trait mainly governed by Zn transporters and metal chelators of plant systems. Notably, previous literature has shown that many transporters, chelators, and key regulatory genes involved in Zn homeostasis simultaneously took part in Fe regulation in plants [33,34,35,36]. To gain a better understanding of Fe and Zn homeostasis in wheat, we recapitulate the extant knowledge of the molecular mechanisms modulating Fe and Zn homeostasis in the model plants *Arabidopsis* and rice. About 172 genes, of which *Arabidopsis* and rice have 110 and 62, respectively, are summarized in Table S1. These provided useful information for the identification of orthologous genes and the dissection of molecular mechanisms underlying Fe and Zn homeostasis in wheat. 

### 2.1. Uptake, Radial Transport, and Loading onto Root Vasculature

To cope with limited Fe availability in soil, plants have evolved two classical strategies to actively take up Fe through the root epidermis. Strategy I is commonly found in non-graminaceous plants (e.g., *Arabidopsis*) and Strategy II, also termed the reducing and chelating strategy, is mainly in graminaceous plants (e.g., rice) [37]. As a Strategy I plant, *Arabidopsis* conducts three steps to acquire Fe at the root–rhizosphere interface. Initially, protons are extruded to the rhizosphere mediated by H^+^-ATPase, causing local rhizosphere acidification and Fe (III) to be more soluble [38]. Subsequently, coumarin family phenolics are exported by the *ABCG37* transporter and chelate solubilized Fe (III), greatly facilitating the Fe (III) acquisition process. In its chelated form, Fe (III) is reduced into Fe (II) by ferric reductase-oxidase 2 (FRO2) located in the plasma membrane [39]. Finally, the resulting Fe (II) is imported into the cell by *iron-regulated transporter 1* (*IRT1*), a member of the *zinc-regulated transporter, iron-regulated transporter-like protein* (*ZIP*) transporter family for divalent metals [40]. 

In Strategy II, graminaceous plants such as wheat and maize utilize the chelation-based strategy to release phytosiderophores (PSs), which are Fe (III)-chelating molecules synthesized in roots. Despite the PSs differing in form among graminaceous species, they are all derived from deoxymugineic acid (DMA), which is synthesized from S-adenosyl-methionine (SAM) through a series of enzymatic reactions. These enzymes include nicotianamine synthase (NAS), nicotianamine aminotranferase (NAAT), and deoxymugineic acid synthase (DMAS) [41,42,43]. The PSs, with high affinity to Fe (III), are then secreted into the rhizosphere mediated by *transporter of mugineic acid family phytosiderophores 1* (*TOM1*) [44]. Once Fe (III)–PS complexes have form in the soil, the mobility of Fe (III) increases and the complexes are then absorbed into the root epidermis by *yellow stripe* (*YS*) or *YS-like* (*YSL*) transporters [45,46,47]. Apart from harnessing Strategy II, rice possesses an IRT-dependent Fe uptake system, which is unique in Strategy I, perhaps as an adaptation to the existence of Fe (II) ions under anaerobic conditions [48,49]. Accumulated evidence showed that the Fe regulon is, to a broader extent, conserved among land plants [50]. Therefore, it is necessary to take account of Strategy I as an Fe acquisition mechanism in wheat. Zn solubilization in soil is mediated by the acidification and secretion of organic chelators (e.g., citrate, malate) in the plant rhizosphere. Nevertheless, the specific contributions of these chelators to plant Zn uptake are still poorly understood. Several *ZIP* family transporters have been proved to contribute to Zn uptake in *Arabidopsis*. Among them, *IRT1-3* is suggested to transport Zn and Fe concurrently [35]. Recently, *OsZIP5* and *OsZIP9* were characterized as the exact transporters synergistically responsible for Zn uptake from soil [51,52].

Prior to arriving at the stele for loading into the vasculature, Fe and Zn have to be radially transferred across different layers of root tissues, including epidermis, cortex, and endodermis. In the symplast, Fe and Zn are combined with chelators to form Fe (II)–NA, Fe (II)–DMA, and Zn–NA complexes to increase transport efficiency. *Metal tolerance protein 2* (*MTP2*) in *Arabidopsis* and *YSL9* in rice are heavily involved in this process [53,54]. In the apoplast space of the xylem, Fe (III)–citrate is the predominant form, and the citrate transporter *ferric reductase detective 3* (*FRD3*) in *Arabidopsis* and its ortholog *FRD-like 1* (*FRDL1*) in rice are thought be crucial for Fe loading into the pericycle cells [55,56,57]. *Ferroportin1* (*FPN1*) in *Arabidopsis* also contributes to loading Fe into the xylem [58]. Zn loading into the xylem is mediated by members of the *heavy metal ATPase* (*HMA*) family of P_1B_-type ATPases, including at least *HMA2* and *HMA9* in rice [59,60]. In *Arabidopsis*, *MTP2* facilitates inward movement of Zn across the root from epidermis to xylem, and *HMA2* then loads the apoplastic xylem with Zn [54]. Additionally, *ZIP4* is possibly involved in phloem Zn loading in rice [61]. 

### 2.2. Xylem and Phloem Transport of Fe and Zn

Once Fe and Zn are loaded into the xylem, they can be transferred to phloem for further long-distance transport and remobilization towards the sink organs. In the acidic xylem (pH = 5.0–6.5), Fe is transported mainly as metal chelates and Zn is in the form of both free ions and metal chelates [62]. Although detailed mechanisms of Fe/Zn xylem-to-phloem transport remain largely unknown, their long-distance transport and remobilization at the source tissues through the phloem are partly revealed. In *Arabidopsis*, *oligo peptide transporter 3* (*OPT3*) contributes to regulating the transportation of a shoot-to-root Fe signal, and redistributing Fe to developing tissues through the phloem [63,64]. In rice, the predominant Fe chelates in the phloem are Fe (II)–NA and Fe (II)–DMA [65]. *OsYSL2* may be related to Fe (II)–NA translocation through the phloem [66,67]. *OsYSL18* and *OsYSL16* are responsible for Fe (II)–DMA translocation via the phloem [68,69,70]. *OsYSL9* transports both Fe (II)–NA and Fe (II)–DMA in the rice phloem sap [53]. In graminaceous plants, stem nodes are of importance as a hub for the distribution of Zn and possibly Fe from xylem to phloem to different above-ground organs [71,72]. *OsZIP3*/*OsHMA2* and *YSL* family transporters/*OsHMA9* are responsible for Zn xylem-to-phloem transfer and phloem-to-organs long-distance transport and remobilization in rice, respectively [59,60,72,73,74]. 

### 2.3. Storage of Fe and Zn in Seeds

After long-distance transport and remobilization, the terminal destination of Fe and Zn in major crops is thought to be seeds. Plants use two major approaches for Fe storage; that is, sequestration into vacuoles and sequestration into ferritin, mainly in the form of ferrous Fe complexes [5]. The proportion of total Fe stored in vacuoles and ferritin differs among plant species [75]. Approximately 50% of the total Fe in *Arabidopsis* seeds is present in vacuolar globoids in endodermal cells, whereas less than 5% is deposited as ferritin [76,77]. Meanwhile, seeds of legumes such as soybean, chick pea, and lentil are especially rich in ferritin. In the grains of cereals such as rice and wheat, Fe is mainly present in vacuoles in the aleurone layer and the seed coat. 

Fe–NA complexes are transported into *Arabidopsis* seeds by *YSL1* and *YSL3* transporters [78,79]. The storage of Fe in seed vacuoles is mediated by *vacuolar iron transporter 1* (*VIT1*) [80]. *NRAMP3* and *NRAMP4* contribute to the export of Fe from the vacuole to the cytosol, an essential step for seed germination [81,82]. *VIT* family genes, including *OsVIT1* and *OsVIT2*, similarly play a crucial role in Fe sequestration into rice vacuoles [83,84], and *NRAMPs* are responsible for Fe transport from the vacuoles [85].

Fe and Zn in wheat are concentrated in small vacuoles in the aleurone layer and sub-aleurone layer, where they localize with phosphorus, most likely in the form of phytic acid (InsP_6_) (PA). Some Fe and Zn are also located in the starchy endosperm [86,87]. Fe and Zn, as complexes with PA, can hardly be assimilated by humans [88]. Hence, a feasible way for wheat biofortification is to simultaneously increase Fe/Zn contents and their bioavailability (e.g., decrease the PA content or increase phytase activity) [87,89]. 

### 2.4. Transcriptional Regulation and Other Factors

An efficient modulation network that senses the status of Fe and Zn in plants, and alters homeostasis accordingly, is vital to ensure that sufficient Fe and Zn reach the appropriate target tissues. Over the past 15 years, substantial numbers of transcription factors (TFs) involved in Fe homeostasis were identified in *Arabidopsis* [90]. *FER-like iron deficiency-induced transcription factor* (*FIT*) is the core transcriptional regulator of Fe homeostasis [91,92]. *FIT* is expressed only in roots and it positively regulates key Fe uptake genes [93]. Various proteins have been identified as interacting directly or indirectly with FIT and an overview of the FIT-regulated network in Fe homeostasis was systematically reviewed by Wu and Ling [94]. Another *bHLH* TF, *POPEYE* (*PYE*), as a negative regulator of *NAS4* and *FRO3* in *Arabidopsis*, also plays an important role in Fe homeostasis independently of *FIT* [95]. Notably, 17 of 133 *bHLH* TFs present in *Arabidopsis* have been reported to regulate Fe homeostasis [90,96]. *MYB* family TFs *MYB10* and *MYB72* participate in regulating Fe acquisition and distribution [97,98,99]. *WRK46* and two *MYB-CC* TFs, *phosphate starvation response 1* (*PHR1*) and *PHR1-like* (*PHL1*), were implicated in Fe translocation and storage, respectively [100,101]. In addition, a small gene family of hemerythrin E3 ubiquitin ligases, such as BRUTUS (BTS), is suggested to sense Fe signals and act as negative regulators of Fe homeostasis [102]. Recently, *BTS-like 1* (*BTSL1*) and *BTS-like 2* (*BTSL2*) were reported to negatively regulate Fe uptake by targeting *FIT* in *Arabidopsis* [103]. A novel peptide family termed *iron man* (*IMA*) or *Fe-uptake-inducing peptide* (*FEP*) positively regulates Fe-deficiency responses in *Arabidopsis* [104,105]. 

TFs governing Fe homeostasis have also been extensively studied in rice [106]. *IRO2* (*iron-deficiency-inducible bHLH TF*) positively modulates PS secretion and the expression of *YSL15*, thereby enhancing Fe uptake [107]. In contrast, *IRO3* negatively regulates the expression of *IRO2* and *NAS*, and inhibits Fe uptake [108]. *ABI3*/*VP1* family *IDEF1* (*iron deficiency-responsive element 1*) and *NAC* family *IDEF2* are both characterized as positive regulators in Fe homeostasis in rice [109,110,111,112]. *OsPRI1* (a positive regulator of iron homeostasis), *OsPRI2* and *OsPRI3*, three *bHLH* TFs identified as direct targets of the hemerythrin motif-containing RING- and zinc-finger proteins (HRZ), induce the expression of *IRO2* and *IRO3* [113,114,115]. *OsbHLH133* is another regulator of Fe distribution between roots and shoots [116]. *OsbHLH156* was recently reported to partner with *IRO*_2_ by localizing *IRO*_2_ to the nucleus [117,118]. 

Several Fe-related transporters and TFs in plants function redundantly and may fine-tune the homeostasis [5]. Some TFs involved in Fe homeostasis can be controlled epigenetically. For instance, *SHK1-binding protein 1* (*SKB1*) catalyzes the symmetric dimethylation of histone H4R3 (H4R3sme2), which in turn suppresses expression of *bHLH38*, *39*, *100*, *and 101* [119]. Meanwhile, thus far, only a few TFs regulating Zn homeostasis have been reported in *Arabidopsis* and rice. *Arabidopsis* TFs *bZIP19* and *bZIP23* regulate several ZIP transporters [120,121]. NA and PS synthesis genes and their regulators involved in Fe homeostasis are also thought to function in Zn homeostasis, due to the important role of NA and PSs in Fe and Zn uptake and transport in plants [23]. 

Apart from TFs, large numbers of plant hormones, small signaling molecules, kinases, and other factors also participate in Fe-deficiency response and Fe homeostasis. Various proteins responsible for the transduction of phytohormones (e.g., ethylene) and signaling molecules (e.g., NO) are able to interplay with FIT/FIT-binding complexes and regulate their activities [122,123]. Ethylene, gibberellin (GA), auxin, and NO positively affect the activity of FIT, whereas cytokinins, abscisic acid (ABA), jasmonic (JA), brassinosteroids (BRs), and H_2_O_2_ suppress Fe-deficiency responses [122,123]. It is worth mentioning that many signal pathways involve cross-talk, and therefore phytohormones and signaling molecules probably influence Fe homeostasis through more than one pathway [124,125]. Recently, Ca^2+^, as a second messenger to transduce environmental cues, was implicated in Fe homeostasis, with calcium B-like interacting protein kinases 23 (CIPK23) and CIPK11 positively regulating Fe-deficiency responses [126,127]. Moreover, *FIT-binding protein* (*FBP*) is involved in the balance of Fe and Zn homeostasis in *Arabidopsis* [128]. In addition, the circadian clock regulator Mediator 16 (MED16) and MED25 modulate Fe uptake by either directly or indirectly interplaying with FIT [129,130]. 

## 3. Genetic Architecture Underlying Fe and Zn Homeostasis in Wheat

Recent advances in sequencing technologies have enabled the development of improved wheat genome assembly and high-throughput genotyping platforms, significantly accelerating forward genetic approaches for wheat gene discovery [131]. Considerable numbers of Fe/Zn-related loci were identified by QTL mapping and genome-wide association studies (GWASs) [132,133]. Simultaneously, reverse genetic approaches such as comparative genomics promote Fe/Zn gene discovery, and some wheat orthologous genes have been cloned recently [134,135]. Here, we used all curated Fe/Zn-related genes from *Arabidopsis* and rice for the identification of wheat orthologs. We summarized the known Fe/Zn-related QTL or genes in wheat and assembled them on wheat chromosomes according to their physical locations. Stable genetic loci for Fe and Zn homeostasis were then identified based on QRCs. 

### 3.1. Identification of Orthologous Wheat Genes

**Figure 1:**
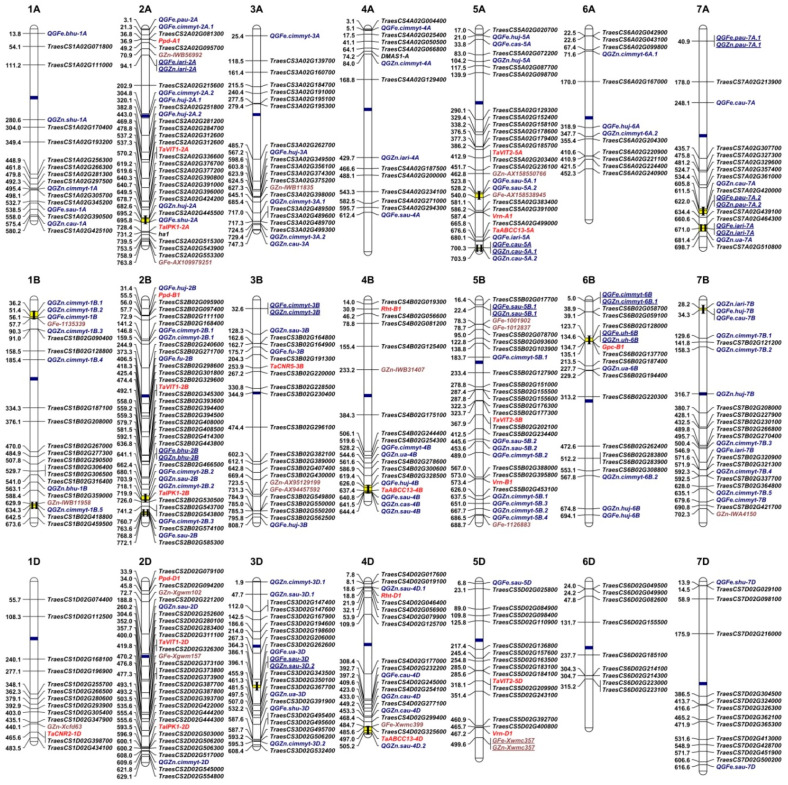
Landscape of genetic architecture underlying Fe- and Zn-related traits in wheat. **1A–7A**, **1B–7B**, and **1D–7D** indicate the numbers of chromosomes in wheat. Genetic loci for Fe and Zn traits identified from wheat orthologs of *Arabidopsis* and rice genes, QTL mapping, association studies, and cloned wheat genes are displayed in black, blue, brown, and red, respectively. Blue and yellow bars within chromosomes indicate the locations of centromeres and QTL-rich clusters. “GFe” and “GZn” indicate grain Fe- and Zn-related loci, respectively. QTL associated with both Fe and Zn are underlined.

In total, 172 Fe/Zn-related gene sequences from *Arabidopsis* and rice were already used in BLAST analysis against the wheat database in EnsemblPlants and 436 wheat orthologous genes were identified (Table S2). Due to the hexaploid nature of common wheat with the A, B, and D sub-genomes, most query genes from model plants have three copies in wheat. Some query genes, mainly from the *FRO*, *YSL*, *FER*, *NARMP*, and *ZIP* gene families have similar gene sequences and correspond to the same wheat genes. After removal of duplications, we finally selected 254 genes with identities greater than 60% for further analysis (Table S3). Orthologous genes were identified on all 21 wheat chromosomes (Figure 1). Among these genes, 82, 86, and 86 were located in the wheat A, B, and D sub-genomes, respectively. Homologous group 2 chromosomes (2A, 2B, and 2D) had the maximum number of genes (58, ~23%), followed by group 5 (39, ~15%) and 3 (36, ~14%), and the minimum number was in group 6 chromosomes (28, ~11%). At the individual chromosome level, 2B had the greatest number of genes (21, ~8%), indicating its significance for Fe/Zn homeostasis.

### 3.2. Cloned Genes for Fe and Zn Homeostasis in Wheat

Prior to the release of the Chinese Spring reference genome sequence, it was relatively difficult to isolate causal genes by map-based cloning in wheat due to its hexaploidy, enormous genome size (~16 Gb), and abundance of repetitive sequences (>85%) [26]. The first Fe/Zn-related gene isolated was *Gpc-B1* derived from *T. turgidum* ssp. *diccocoides* and characterized as *NAM-B1*, a *NAC* family TF. It accelerates senescence and increases remobilization of Fe, Zn, and nitrogen compounds from source organs to the grain [136,137,138]. The frequency of the functional allele is rare in modern Australian cultivars [139] and zero in Chinese wheats [140]. Introgression of the functional *Gpc-B1* allele improved wheat grain Fe and Zn content, but tended to cause some undesirable traits, such as senescence, reduced panicle number, and low grain weight [137,141,142,143,144].

Comparative genomics provides an efficient way to identify and clone orthologous genes with conserved functions [145]. Six genes representing 14 loci have been characterized to affect wheat grain Fe/Zn (Table S4; Figure 1). By homologous cloning, two *VIT* paralogs, *TaVIT1* and *TaVIT2*, were identified in wheat, and occur as three copies on wheat group 2 and 5 chromosomes, respectively [146]. *TaVIT2* is responsible for vacuolar Fe transport in the endosperm and was confirmed to be effective in wheat biofortification [146]. Cell number regulator 2 (TaCNR2) and TaCNR5 identified in wheat contain similar conserved domains as plant cadmium resistance proteins (PCR) [134,135]. Furthermore, *TaCNR2* and *TaCNR5* were characterized to affect heavy metal transport and accumulation in plant seeds [134,135]. In addition, wheat ABCC transporter *TaABCC13* and inositol pentakisphosphate kinase (*TaIPK1*), with copies in each sub-genome, were reported to be involved in PA biosynthesis [147,148,149]. RNAi-mediated downregulation of *TaABCC13* caused reduced PA without significantly affecting Fe and Zn concentrations in wheat grains, whereas silencing of *TaIPK1* significantly decreased PA levels while enhancing grain Fe and Zn contents [148,149].

Notably, “Green Revolution” genes *Rht-B1b* and *Rht-D1b* not only reduced plant height and influence grain yield, but also had negative effects on grain micronutrient concentrations in wheat [17,19,150,151,152,153,154]. Additionally, the photoperiod insensitivity alleles (*Ppd1*) and possibly flowering time regulatory alleles (*Vrn1*) were associated with wheat micronutrient concentration, indicating that the development timing also affects micronutrient traits (Table S4) [155]. Doubtlessly, more Fe/Zn-related genes are yet to be cloned and their allelic variations affecting gene expression need to be determined. The development of gene-based markers will greatly benefit marker-assisted selection of Fe/Zn-enriched wheat [27,156].

### 3.3. Genetic Loci Detected by Linkage Mapping and Association Mapping in Wheat

About 120 Fe/Zn-related QTL have been mapped on most wheat chromosomes (Table S5) [132,141,157,158,159,160,161,162,163,164,165,166,167,168,169,170,171,172,173]. These are shown in Figure 1, using the physical locations of the closest linked markers or midpoints of the respective genetic intervals. In addition, more than 20 relatively stable genetic loci for Fe or/and Zn have been reported from GWASs (Table S5; Figure 1) [133,174,175,176,177,178,179]. Among them, three Fe/Zn loci match well with those reported from QTL linkage mapping, indicating a degree of reliability. When all the genetic loci detected by QTL mapping and GWASs were placed in Figure 1, the distribution patterns at the chromosome level were different. Among the three wheat sub-genomes, B had the largest number (67) of Fe/Zn-related QTL, followed by A (49), and D (24). Group 2 chromosomes had the largest number (26), whereas group 6 chromosomes had the least (11). These findings were partially consistent with the distribution patterns of orthologous genes as mentioned above, and again indicated that group 2 chromosomes carried multiple genes affecting Fe/Zn homeostasis, whereas the contributions by group 6 chromosomes were relatively minor. It was also not unexpected to find 12 pleiotropic QTL associated with Fe and Zn because the same transporters and chelators, such as IRT1 and NA, are shared for both Fe and Zn homeostasis. 

### 3.4. Reliable Genetic Loci for Fe and Zn Homeostasis Based on QTL-Rich Clusters (QRCs) 

Fe and Zn homeostasis in plants are complex traits controlled by a great number of QTL and characterized by large effect of genotype × environment interactions [180]. These environmental factors mainly include cross-talk between Fe/Zn and other cations, including carbon (C), nitrogen (N), phosphorus (P), and potassium (K); different environmental conditions, such as soil type, water, and future climatic changes; and field management, such as foliar or soil Fe/Zn fertilization [31,181]. Rational N fertilization increases grain Zn content by N–Zn synergism, whereas P input generally decreases Zn by P–Zn antagonism [181]. Increasing Zn supply to wheat by soil or foliar fertilization can effectively improve Zn concentrations in grains [182]. Considering the large impact of environment factors and genotype × environment interactions on Fe/Zn phenotypes, it is appropriate to conduct multi-environment experiments in fields with sufficient Fe/Zn fertilization for mining Fe/Zn genetic loci. Near isogenic lines (NILs) are ideal materials to reduce confounded interactions of genotype × environment and to investigate the reliable loci [137].

The inheritance of Fe/Zn traits is frequently disrupted by epistatic interactions among multiple QTL/genes [168]. However, the mapping of genetic loci for Fe/Zn traits to the same or close chromosome regions in different studies is indicative of true and stable genetic effects. To identify such regions, we defined QRCs as the regions of <10 Mb including two or more QTL for the same trait from different studies [183]. We investigated distributions of Fe/Zn QTL across chromosomes and identified 14 QRCs based on the above criteria (Table 1; Figure 1). Only four QRCs were specific for Fe or Zn, and other ten were related to both Fe and Zn, indicating that these QRCs may harbor the same or very closely linked candidate genes for Fe and Zn homeostasis. Considering the 12 QTL associated with both Fe and Zn described above, further fine mapping and isolation of functional genes located in these QRCs will benefit the understanding of molecular mechanisms underlying Fe/Zn traits in wheat. The chromosomal distributions of QRCs show that five are located in sub-genome A, and seven are located in sub-genome B, whereas only two are in D. Wheat sub-genome D tends to harbor limited molecular markers so that Fe/Zn loci are identified much less often than those in sub-genomes A and B. However, surprisingly, wheat orthologs of genes for Fe/Zn are found to be abundant in the D sub-genome (86 out of 254), suggesting potential to exploit Fe/Zn genes on the D sub-genome and to further investigate their polymorphisms. In comparison with other agronomic traits, such as yield or disease resistance, the number of studies on Fe and Zn is much smaller and identification of related QRCs is relatively limited [183,184].

Many wheat orthologs (41, ~15%) identified in this study were closely (<10 Mb) linked with Fe/Zn QTL discovered in the linkage and association mapping (Figure 1), indicating their probable candidate gene roles. Once validated, these genes could be immediately implemented in breeding for biofortification. Orthologous genes not linked with known QTL are probably monomorphic in most wheat cultivars, or a QTL has not yet been identified in those regions (Figure 1). Conversely, the many individual QTL found in the regions without known candidate genes might represent unique genes in wheat. Isolation of these genes and dissection of their molecular mechanisms will be extremely important for wheat biofortification. Using GWAS and QTL mapping approaches, we recently identified 20 new Fe/Zn loci, mostly from Chinese wheat genotypes, and seven of them were very close to candidate orthologs. One candidate gene on chromosome 3AL, as an ortholog of *OsPEZ*, an Fe uptake-related gene, is worthy of further validation. We also identified 15 loci that were probably the same as the known QTL, and at least eight have gene candidates mapped as shown in Figure 1 (our unpublished data). Thus, the current study provides a roadmap for the continuous discovery of wheat Fe/Zn-related genes. 

## 4. Genetic Biofortification to Enrich Grain Fe and Zn in Wheat

**Figure 2:**
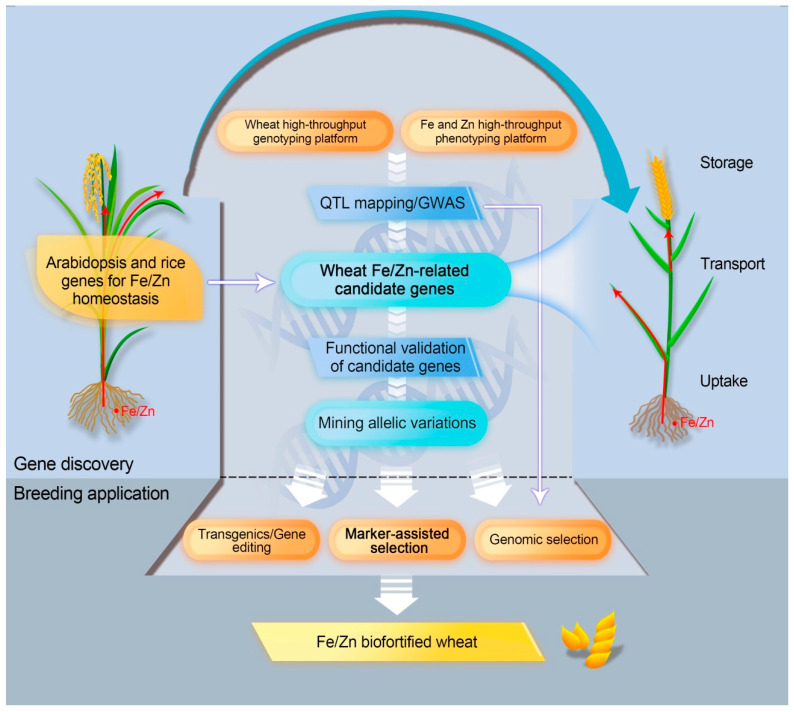
Schematic diagram of genetic biofortification strategies to enrich Fe and Zn in wheat grain.

Conventional breeding has attained few successes in enriching Fe and Zn in wheat grain. Biofortification harnessing molecular biotechnology and genetic engineering promises a multi-dimensional avenue to enhance the required micronutrients in the grains [185,186,187]. Using orthologs of Fe/Zn homeostasis genes from model plants and altering their expression in staple crops have offered valuable insights into genetic biofortification. Nevertheless, in comparison to other staple crops (e.g., rice and maize), the practice has appeared to be less to develop Fe/Zn-rich grains in wheat using transgenic and biotechnology approaches [87,146,149,188,189,190]. Here, we emphasize different research paths to leverage genetic architecture for Fe/Zn, which enable fast release of Fe/Zn-biofortified wheats (Figure 2).

### 4.1. Transgene and Gene Editing Approaches 

The 172 genes from *Arabidopsis* and rice for Fe/Zn homeostasis are mainly involved in Fe/Zn uptake, translocation, storage, and regulation. In the process of Fe/Zn uptake, ferric reductases, proton ATPases, and NA/DMA synthases play key roles, and the corresponding candidate genes are to be proved and used for increasing plant Fe/Zn contents. For Fe/Zn translocation, many key transporters, such as *FRD3*, *FPN1*, the *ZIP* family, and the *YSL* family, are predominantly responsible for homeostatic step. In wheat, Fe and Zn are mainly deposited in the aleurone layer, whereas some Fe and Zn are also located in the starchy endosperm [86,87]. Hence, whole-grain flour has a higher amount of Fe and Zn than white flour, but improved Fe and Zn could also be achieved in white flour by using key genes/proteins, including ferritin, *VIT*, and the *NRAMP* family, which are expressed in endosperm. Alternatively, specific TFs and other Fe/Zn regulation factors can also be considered as potential target genes for biofortification as long as they confer no or little yield penalty.

In principle, any of the above proteins/genes can be a target for biofortification. The transformation of individual or combined key Fe/Zn-related genes from model plants to wheat is demonstrated to be an effective strategy for Fe/Zn biofortification in wheat. A successful example was overexpression of *OsNAS2* or/and *PvFERRITIN* in wheat, which has seen significantly higher Fe concentration of grains compared with the control [87,190]. Another case was that enriched grain Fe content was achieved by overexpression of wheat *FERRITIN* and *TaVIT2* [146,189].

Other than the transgene approach, gene editing could be a more powerful tool to make predicted changes in target genes by deactivating, generating functional alleles, replacing mutant alleles, or creating site-specific transgene integration [191]. Despite a wide range of applications (e.g., yield traits and pre-harvest sprouting resistance) already reported in wheat genome editing, there is no report for editing in Fe/Zn-related genes [192,193]. Gene editing tools, especially the emerging plant prime editing (PPE), enable precise gene modification, including single base substitution and multiple base insertion/deletion, and thus are able to offer potential for wheat Fe/Zn biofortification [194,195]. *Gpc-B1* could serve as a starting point for candidate gene editing since removal of 1-bp in the exon in certain wheat genotypes can recover the expected function of *NAC*, as higher content of Fe/Zn was detected in grains. 

### 4.2. Marker-Assisted Selection (MAS) and Genomic Selection

Although transgene and gene editing approaches can effectively and precisely increase intrinsic grain Fe/Zn content, their applications are seriously restricted by public acceptance and government regulations [196]. Marker-assisted selection is dependent on the inheritance of a high-Fe/Zn phenotype, together with a particular genetic marker, and has been widely used in wheat breeding. In the previous section, we summarized, in total, 140 wheat Fe/Zn-related genetic loci detected by linkage mapping and association mapping, as well as 14 QRCs defined as stable and reliable Fe/Zn-related clusters. Close linkage of markers for these genetic loci can be further confirmed irrespective of whether marker–trait associations were outcomes of bi-parental hybrid populations or large natural populations. Thereafter, the confirmed molecular markers, or ideally functional markers for causal genes, will be used in marker-assisted breeding for high Fe and Zn. *Gpc-B1*, as an example, has been exploited for wheat grain Fe/Zn enhancement following marker-assisted breeding. Interestingly, most lines carrying *Gpc-B1* showed significantly higher Fe and Zn contents [197]. Instead of incorporating only significant markers into a selection model in MAS breeding, genomic selection (GS) uses genomically estimated breeding values (GEBVs) developed by all markers covering the entire genome. Similar to MAS, GS can be carried out at any location, including non-target environments so that the breeding process can be greatly accelerated at lower cost [198]. GS could serve as a potential method for Fe/Zn biofortification but one important factor affecting GS accuracy is whether the markers of key QTL/genes are incorporated in the model as a fixed effect [199]. Therefore, a continuing effort in Fe/Zn gene discovery and marker development will not only facilitate breeding for biofortification but will also improve the accuracy of molecular tools for future wheat improvement.

### 4.3. Fast Tracking the Use of Orthologous Genes in Wheat Genetics and Breeding

In this review, 254 Fe/Zn-related gene orthologs were identified in the wheat genome. Utilizing genotyping by targeted sequencing (GBTS), a targeted sequence-capture strategy, these genes can be amplified and sequenced for identification of polymorphic sites in large populations with natural variations, followed by verification of associations with wheat Fe/Zn traits [27,200,201]. Orthologous genes closely linked to known QTL can be given priority. Additionally, the orthologous wheat genes can be functionally validated using reverse genetics approaches, such as heterologous expression, silencing, or knockout, and overexpression [202]. It is interesting that some orthologous genes (e.g., *TraesCS2D02G373800* and *TraesCS2D02G373900* on chromosome 2D) are present as tandem duplications, since a recent study reported that some copy number variations (CNVs) may be important in wheat stress resilience [203]. Polymorphism in CNVs cannot be detected using SNP chips, thus these genes might not be identified despite potential regulatory functions in quantitative traits. GBTS or gene expression profiling could reveal such polymorphisms. 

The described wheat Fe/Zn orthologous genes are potentially related to Fe and Zn homeostasis, and provide valuable resources for marker-assisted selection and/or for gene manipulation. The increasing identification/characterization of Fe/Zn-related genes will promote the elucidation of regulatory mechanisms underlying Fe/Zn homeostasis of wheat. Moreover, the discovery of potential genes with pleiotropic effects on improving both nutrients and yield is of great significance for breeding more nutritious wheats with high yield, and potentially higher economic returns for farmers.

## 5. Conclusions and Future Prospects

The pace of genetic and genomics research has accelerated with the availability of increasing amounts of genomics information for common wheat. The current review exhibits the molecular processes and genetic landscape of Fe and Zn traits through referential and comparative molecular mechanisms from model plants, provides the first inventory genes or loci regulating wheat grain Fe and Zn homeostasis from diverse approaches, and identifies reliable genetic loci based on the fully annotated wheat reference genome. Molecular marker technology will be an efficient tool for breeding Fe/Zn-biofortified cultivars. Allelic variations of substantial numbers of Fe and Zn candidate genes need to be detected, and the development and establishment of high-throughput and cost-effective genotyping platforms (e.g., GBTS genotyping platform) are of great importance for practical high-Fe and -Zn breeding. The screening and pyramiding of superior allelic variants will be the next stage of development for wheat Fe/Zn biofortification. However, it is unsuitable to pyramid too many high-Fe and -Zn genes simultaneously, since the latent metal toxicity is detrimental to wheat growth. In model plants, a large number of transporters, TFs, plant hormones, small signaling molecules, kinases, and other factors were revealed to be involved in Fe/Zn homeostasis. The understanding of the above factors and regulatory mechanisms is providing resources to elucidate the molecular regulatory networks in wheat with the current limited molecular progress. Multi-omics methods (e.g., genomics, metabolomics, transcriptomics, and bioinformatics) and emerging biotechnologies will promote the deciphering of the mechanisms regulating Fe/Zn uptake, translocation, and storage in wheat, contributing to the application of transgene and gene editing approaches in breeding programs.

## Figures and Tables

**Table 1:** QTL-rich clusters (QRCs) for wheat Fe and Zn traits.

QRC	Interval (Mb) ^a^	Included Genetic Locus ^b^	Trait ^c^
1B-I	36.2–57.7	*QGZn.cimmyt-1B.1*, *QGZn.cimmyt-1B.2*, *QGFe.cimmyt-1B*, *GFe-1135339*	GFe, GZn
1B-II	629.9–642.5	*GZn-IWB11958*, *QGZn.cimmyt-1B.5*, *TraesCS1B02G418800*	GZn
2A-I	678.7–695.2	*TraesCS2A02G424200*, *QGZn.huj-2A*, *TraesCS2A02G445500*, *QGFe.shu-2A*	GFe, GZn
2B-I	703.9–719.9	*QGZn.sau-2B*, *QGZn.cimmyt-2B.2*, *TaIPK1-2B*	GZn
2B-II	760.7–772.1	*QGFe.cimmyt-2B.3*, *TraesCS2B02G574100*, *QGFe.sau-2B*, *TraesCS2B02G585300*	GFe
3D-I	386.1–396.1	*QGFe.tua-3D*, *QGFe.sau-3D*, *QGZn.sau-3D.2*	GFe, GZn
4B-I	619.4–644.4	*TraesCS4B02G328500*, *QGFe.huj-4B*, *TaABCC13-4B*, *QGFe.sau-4B*, *QGZn.cas-4B*, *QGZn.sau-4B*	GFe, GZn
4D-I	484.7–505.2	*GFe-Xwmc399*, *TraesCS4D02G325600*, *TaABCC13-4D*, *QGZn.sau-4D.2*	GFe, GZn
5A-I	523.8–540.0	*QGFe.sau-5A.1*, *QGFe.sau-5A.2*, *GFe-AX158538945*	GFe
5A-II	700.3–703.9	*QGFe.cau-5A*, *QGZn.cau-5A.1*, *QGZn.cau-5A.2*	GFe, GZn
6B-I	123.7–135.1	*TraesCS6B02G128000*, *QGFe.uh-6B*, *QGZn.uh-6B*, *Gpc-B1, TraesCS6B02G137700*	GFe, GZn
7A-I	605.8–622.0	*QGZn.cau-7A*, *TraesCS7A02G420000*, *QGFe.pau-7A.2*, *QGZn.pau-7A.2*	GFe, GZn
7A-II	660.6–681.4	*TraesCS7A02G464300*, *QGFe.iari-7A*, *QGZn.iari-7A*, *QGZn.ua-7A*	GFe, GZn
7B-I	28.2–34.3	*QGZn.iari-7B*, *QGFe.cau-7B*, *QGFe.huj-7B*	GFe, GZn

^a^ Intervals of QTL-rich clusters (QRCs) were defined according to IWGSC RefSeq v1.0. ^b^ Some loci were designated in this study because they were not named in the references. Cloned genes in QRCs are underlined. ^c^ “GFe” and “GZn” represent grain Fe concentration and grain Zn concentration, respectively.

## Supplementary Materials

The following are available online at https://www.mdpi.com/1422-0067/21/23/9280/s1, Table S1: 172 *Arabidopsis* and rice genes for Fe and Zn traits. Table S2: 436 unfiltered wheat orthologs of *Arabidopsis* and rice genes for Fe and Zn traits. Table S3: 254 filtered wheat orthologs of *Arabidopsis* and rice genes for Fe and Zn traits. Table S4: 11 cloned genes for wheat Fe and Zn traits. Table S5: 140 quantitative trait loci detected by linkage mapping or association analyses for wheat Fe and Zn traits.

## References

[1] Hänsch R., Mendel R.R. (2009). Physiological functions of mineral micronutrients (Cu, Zn, Mn, Fe, Ni, Mo, B, Cl). Curr. Opin. Plant Biol..

[2] Zhang Y., Gladyshev V.N. (2011). Comparative genomics of trace element dependence in biology. J. Biol. Chem..

[3] Garcia-Oliveira A.L., Chander S., Ortiz R., Menkir A., Gedil M. (2018). Genetic basis and breeding perspectives of grain iron and zinc enrichment in cereals. Front. Plant Sci..

[4] Schmidt W. (2003). Iron solutions: Acquisition strategies and signaling pathways in plants. Trends Plant Sci..

[5] Connorton J.M., Balk J., Rodríguez-Celma J. (2017). Iron homeostasis in plants-a brief overview. Metallomics.

[6] Bailey S., Thompson E., Nixon P.J., Horton P., Mullineaux C.W., Robinson C., Mann N.H. (2002). A critical role for the Var2 FtsH homologue of *Arabidopsis thaliana* in the photosystem II repair cycle in vivo. J. Biol. Chem..

[7] Lin C.W., Chang H.B., Huang H.J. (2005). Zinc induces mitogen-activated protein kinase activation mediated by reactive oxygen species in rice roots. Plant Physiol. Biochem..

[8] Zhang X., Zhang D., Sun W., Wang T. (2019). The adaptive mechanism of plants to iron deficiency via iron uptake, transport, and homeostasis. Int. J. Mol. Sci..

[9] Gupta N., Ram H., Kumar B. (2016). Mechanism of zinc absorption in plants: Uptake, transport, translocation and accumulation. Rev. Environ. Sci. Bio/Technol..

[10] Vasconcelos M.W., Gruissem W., Bhullar N.K. (2017). Iron biofortification in the 21st century: Setting realistic targets, overcoming obstacles, and new strategies for healthy nutrition. Curr. Opin. Biotechnol..

[11] Stevens G.A., Finucane M.M., De-Regil L.M., Paciorek C.J., Flaxman S.R., Branca F., Peña-Rosas J.P., Bhutta Z.A., Ezzati M. (2013). Global, regional, and national trends in haemoglobin concentration and prevalence of total and severe anaemia in children and pregnant and non-pregnant women for 1995–2011: A systematic analysis of population-representative data. Lancet Glob. Health.

[12] Lopez A., Cacoub P., Macdougall I.C., Peyrin-Biroulet L. (2016). Iron deficiency anaemia. Lancet.

[13] Stein A.J., Meenakshi J.V., Qaim M., Nestel P., Sachdev H.P.S., Bhutta Z.A. Health benefits of biofortification: An ex-ante analysis of iron-rich rice and wheat in India. Proceedings of the American Agricultural Economics Association Annual Meeting.

[14] Gómez-Galera S., Rojas E., Sudhakar D., Zhu C., Pelacho A.M., Capell T., Christou P. (2010). Critical evaluation of strategies for mineral fortification of staple food crops. Transgenic Res..

[15] Ludwig Y., Slamet-Loedin I.H. (2019). Genetic biofortification to enrich rice and wheat grain iron: From genes to product. Front. Plant Sci..

[16] Monasterio I., Graham R.D. (2000). Breeding for trace minerals in wheat. Food Nutr. Bull..

[17] Fan M.S., Zhao F.J., Fairweather-Tait S.J., Poulton P.R., Dunham S.J., McGrath S.P. (2008). Evidence of decreasing mineral density in wheat grain over the last 160 years. J. Trace Elem. Med. Biol..

[18] Murphy K.M., Reeves P.G., Jones S.S. (2008). Relationship between yield and mineral nutrient concentrations in historical and modern spring wheat cultivars. Euphytica.

[19] Shewry P.R., Pellny T.K., Lovegrove A. (2016). Is modern wheat bad for health?. Nat. Plants.

[20] Davis D.R. (2009). Declining fruit and vegetable nutrient composition: What is the evidence?. HortScience.

[21] Graham R.D., Knez M., Welch R.M. (2012). How much nutritional iron deficiency in humans globally is due to an underlying zinc deficiency?. Adv. Agron..

[22] Kobayashi T., Nishizawa N.K. (2012). Iron uptake, translocation, and regulation in higher plants. Annu. Rev. Plant Biol..

[23] Sinclair S.A., Krämer U. (2012). The zinc homeostasis network of land plants. Biochim. Biophys. Acta Mol. Cell Res..

[24] Bonneau J., Baumann U., Beasley J., Li Y., Johnson A.A.T. (2016). Identification and molecular characterization of the nicotianamine synthase gene family in bread wheat. Plant Biotechnol. J..

[25] Kumar A., Kaur G., Goel P., Bhati K.K., Kaur M., Shukla V., Pandey A.K. (2019). Genome-wide analysis of oligopeptide transporters and detailed characterization of yellow stripe transporter genes in hexaploid wheat. Funct. Integr. Genom..

[26] Appels R., Eversole K., Stein N., Feuillet C., Keller B., Rogers J., Pozniak C.J., Choulet F., Distelfeld A., Poland J. (2018). Shifting the limits in wheat research and breeding using a fully annotated reference genome. Science.

[27] Rasheed A., Hao Y., Xia X., Khan A., Xu Y., Varshney R.K., He Z. (2017). Crop breeding chips and genotyping platforms: Progress, challenges, and perspectives. Mol. Plant.

[28] Velu G., Crespo Herrera L., Guzman C., Huerta J., Payne T., Singh R.P. (2019). Assessing genetic diversity to breed competitive biofortified wheat with enhanced grain Zn and Fe concentrations. Front. Plant Sci..

[29] Kopittke P.M., Punshon T., Paterson D.J., Tappero R.V., Wang P., Blamey F.P.C., van der Ent A., Lombi E. (2018). Synchrotron-based X-ray fluorescence microscopy as a technique for imaging of elements in plants. Plant Physiol..

[30] Paltridge N.G., Milham P.J., Ortiz-Monasterio J.I., Velu G., Yasmin Z., Palmer L.J., Guild G.E., Stangoulis J.C.R. (2012). Energy-dispersive X-ray fluorescence spectrometry as a tool for zinc, iron and selenium analysis in whole grain wheat. Plant Soil.

[31] Clemens S. (2019). Metal ligands in micronutrient acquisition and homeostasis. Plant Cell Environ..

[32] Andresen E., Peiter E., Küpper H. (2018). Trace metal metabolism in plants. J. Exp. Bot..

[33] Bashir K., Takahashi R., Nakanishi H., Nishizawa N.K. (2013). The road to micronutrient biofortification of rice: Progress and prospects. Front. Plant Sci..

[34] Masuda H., Aung M.S., Nishizawa N.K. (2013). Iron biofortification of rice using different transgenic approaches. Rice.

[35] Shanmugam V., Lo J.C., Yeh K.C. (2013). Control of Zn uptake in *Arabidopsis halleri*: A balance between Zn and Fe. Front. Plant Sci..

[36] Page V., Feller U. (2015). Heavy metals in crop plants: Transport and redistribution processes on the whole plant level. Agronomy.

[37] Römheld V., Marschner H. (1986). Evidence for a specific uptake system for iron phytosiderophores in roots of grasses. Plant Physiol..

[38] Santi S., Schmidt W. (2009). Dissecting iron deficiency-induced proton extrusion in Arabidopsis roots. New Phytol..

[39] Robinson N.J., Procter C.M., Connolly E.L., Guerinot M.L. (1999). A ferric-chelate reductase for iron uptake from soils. Nature.

[40] Eide D., Broderius M., Fett J., Guerinot M.L. (1996). A novel iron-regulated metal transporter from plants identified by functional expression in yeast. Proc. Natl. Acad. Sci. USA.

[41] Higuchi K., Suzuki K., Nakanishi H., Yamaguchi H., Nishizawa N.-K., Mori S. (1999). Cloning of nicotianamine synthase genes, novel genes involved in the biosynthesis of phytosiderophores. Plant Physiol..

[42] Takahashi M., Yamaguchi H., Nakanishi H., Shioiri T., Nishizawa N.-K., Mori S. (1999). Cloning two genes for nicotianamine aminotransferase, a critical enzyme in iron acquisition (Strategy II) in graminaceous plants. Plant Physiol..

[43] Bashir K., Inoue H., Nagasaka S., Takahashi M., Nakanishi H., Mori S., Nishizawa N.K. (2006). Cloning and characterization of deoxymugineic acid synthase genes from graminaceous plants. J. Biol. Chem..

[44] Nozoye T., Nagasaka S., Kobayashi T., Takahashi M., Sato Y., Sato Y., Uozumi N., Nakanishi H., Nishizawa N.K. (2011). Phytosiderophore efflux transporters are crucial for iron acquisition in graminaceous plants. J. Biol. Chem..

[45] Murata Y., Ma J.F., Yamaji N., Ueno D., Nomoto K., Iwashita T. (2006). A specific transporter for iron(III)-phytosiderophore in barley roots. Plant J..

[46] Inoue H., Kobayashi T., Nozoye T., Takahashi M., Kakei Y., Suzuki K., Nakazono M., Nakanishi H., Mori S., Nishizawa N.K. (2009). Rice OsYSL15 is an iron-regulated iron (III)-deoxymugineic acid transporter expressed in the roots and is essential for iron uptake in early growth of the seedlings. J. Biol. Chem..

[47] Lee S., Chiecko J.C., Kim S.A., Walker E.L., Lee Y., Guerinot M.L., An G. (2009). Disruption of *OsYSL15* leads to iron inefficiency in rice plants. Plant Physiol..

[48] Ishimaru Y., Suzuki M., Tsukamoto T., Suzuki K., Nakazono M., Kobayashi T., Wada Y., Watanabe S., Matsuhashi S., Takahashi M. (2006). Rice plants take up iron as an Fe^3+^-phytosiderophore and as Fe^2+^. Plant J..

[49] Liu C., Gao T., Liu Y., Liu J., Li F., Chen Z., Li Y., Lv Y., Song Z., Reinfelder J.R. (2019). Isotopic fingerprints indicate distinct strategies of Fe uptake in rice. Chem. Geol..

[50] Grillet L., Schmidt W. (2019). Iron acquisition strategies in land plants: Not so different after all. New Phytol..

[51] Huang S., Sasaki A., Yamaji N., Okada H., Mitani-Ueno N., Ma J.F. (2020). The ZIP transporter family member OsZIP9 contributes to root zinc uptake in rice under zinc-limited conditions. Plant Physiol..

[52] Tan L., Qu M., Zhu Y., Peng C., Wang J., Gao D., Chen C. (2020). ZINC TRANSPORTER5 and ZINC TRANSPORTER9 function synergistically in zinc/cadmium uptake. Plant Physiol..

[53] Senoura T., Sakashita E., Kobayashi T., Takahashi M., Aung M.S., Masuda H., Nakanishi H., Nishizawa N.K. (2017). The iron-chelate transporter OsYSL9 plays a role in iron distribution in developing rice grains. Plant Mol. Biol..

[54] Sinclair S.A., Senger T., Talke I.N., Cobbett C.S., Haydon M.J., Krämer U. (2018). Systemic upregulation of MTP2- and HMA2-mediated Zn partitioning to the shoot supplements local Zn deficiency responses. Plant Cell.

[55] Durrett T.P., Gassmann W., Rogers E.E. (2007). The FRD3-mediated efflux of citrate into the root vasculature is necessary for efficient iron translocation. Plant Physiol..

[56] Green L.S., Rogers E.E. (2004). *FRD3* controls iron localization in Arabidopsis. Plant Physiol..

[57] Yokosho K., Yamaji N., Ma J.F. (2016). *OsFRDL1* expressed in nodes is required for distribution of iron to grains in rice. J. Exp. Bot..

[58] Morrissey J., Baxter I.R., Lee J., Li L., Lahner B., Grotz N., Kaplan J., Salt D.E., Guerinot M. (2009). Lou The ferroportin metal efflux proteins function in iron and cobalt homeostasis in *Arabidopsis*. Plant Cell.

[59] Lee S., Kim Y.Y., Lee Y., An G. (2007). Rice P_1B_-type heavy-metal ATPase, OsHMA9, is a metal efflux protein. Plant Physiol..

[60] Yamaji N., Xia J., Mitani-Ueno N., Yokosho K., Ma J.F. (2013). Preferential delivery of zinc to developing tissues in rice is mediated by P-type heavy metal ATPase OsHMA2. Plant Physiol..

[61] Ishimaru Y., Suzuki M., Kobayashi T., Takahashi M., Nakanishi H., Mori S., Nishizawa N.K. (2005). OsZIP4, a novel zinc-regulated zinc transporter in rice. J. Exp. Bot..

[62] Yoneyama T., Ishikawa S., Fujimaki S. (2015). Route and regulation of zinc, cadmium, and iron transport in rice Plants (*Oryza sativa* L.) during vegetative growth and grain filling: Metal transporters, metal speciation, grain Cd reduction and Zn and Fe biofortification. Int. J. Mol. Sci..

[63] Zhai Z., Gayomba S.R., Jung H.I., Vimalakumari N.K., Piñeros M., Craft E., Rutzke M.A., Danku J., Lahner B., Punshon T. (2014). OPT3 is a phloem-specific iron transporter that is essential for systemic iron signaling and redistribution of iron and cadmium in *Arabidopsis*. Plant Cell.

[64] Mendoza-Cózatl D.G., Xie Q., Akmakjian G.Z., Jobe T.O., Patel A., Stacey M.G., Song L., Demoin D.W., Jurisson S.S., Stacey G. (2014). OPT3 is a component of the iron-Signaling network between leaves and roots and misregulation of *OPT3* leads to an over-accumulation of cadmium in seeds. Mol. Plant.

[65] Rellán-Álvarez R., Abadía J., Álvarez-Fernández A. (2008). Formation of metal-nicotianamine complexes as affected by pH, ligand exchange with citrate and metal exchange. A study by electrospray ionization time-of-flight mass spectrometry. Rapid Commun. Mass Spectrom..

[66] Ishimaru Y., Masuda H., Bashir K., Inoue H., Tsukamoto T., Takahashi M., Nakanishi H., Aoki N., Hirose T., Ohsugi R. (2010). Rice metal-nicotianamine transporter, OsYSL2, is required for the long-distance transport of iron and manganese. Plant J..

[67] Koike S., Inoue H., Mizuno D., Takahashi M., Nakanishi H., Mori S., Nishizawa N.K. (2004). OsYSL2 is a rice metal-nicotianamine transporter that is regulated by iron and expressed in the phloem. Plant J..

[68] Aoyama T., Kobayashi T., Takahashi M., Nagasaka S., Usuda K., Kakei Y., Ishimaru Y., Nakanishi H., Mori S., Nishizawa N.K. (2009). OsYSL18 is a rice iron (III)-deoxymugineic acid transporter specifically expressed in reproductive organs and phloem of lamina joints. Plant Mol. Biol..

[69] Kakei Y., Ishimaru Y., Kobayashi T., Yamakawa T., Nakanishi H., Nishizawa N.K. (2012). OsYSL16 plays a role in the allocation of iron. Plant Mol. Biol..

[70] Lee S., Ryoo N., Jeon J.-S., Guerinot M.L., An G. (2012). Activation of rice *Yellow Stripe1-Like 16* (*OsYSL16*) enhances iron efficiency. Mol. Cells.

[71] Tsukamoto T., Nakanishi H., Uchida H., Watanabe S., Matsuhashi S., Mori S., Nishizawa N.K. (2009). Fe translocation in barley as monitored by a positron-emitting tracer imaging system (PETIS): Evidence for the direct translocation of Fe from roots to young leaves via phloem. Plant Cell Physiol..

[72] Yamaji N., Ma J.F. (2014). The node, a hub for mineral nutrient distribution in graminaceous plants. Trends Plant Sci..

[73] Curie C., Cassin G., Couch D., Divol F., Higuchi K., Le Jean M., Misson J., Schikora A., Czernic P., Mari S. (2009). Metal movement within the plant: Contribution of nicotianamine and yellow stripe 1-like transporters. Ann. Bot..

[74] Sasaki A., Yamaji N., Mitani-Ueno N., Kashino M., Ma J.F. (2015). A node-localized transporter OsZIP3 is responsible for the preferential distribution of Zn to developing tissues in rice. Plant J..

[75] Zielińska-Dawidziak M. (2015). Plant ferritin-a source of iron to prevent its deficiency. Nutrients.

[76] Ravet K., Touraine B., Kim S.A., Cellier F., Thomine S., Guerinot M.L., Briat J.F., Gaymard F. (2009). Post-translational regulation of AtFER2 ferritin in response to intracellular iron trafficking during fruit development in *Arabidopsis*. Mol. Plant.

[77] Roschzttardtz H., Conéjéro G., Curie C., Mari S. (2009). Identification of the endodermal vacuole as the iron storage compartment in the Arabidopsis embryo. Plant Physiol..

[78] Waters B.M., Chu H.-H., DiDonato R.J., Roberts L.A., Eisley R.B., Lahner B., Salt D.E., Walker E.L. (2006). Mutations in Arabidopsis *Yellow Stripe-Like1* and *Yellow Stripe-Like3* reveal their roles in metal ion homeostasis and loading of metal ions in seeds. Plant Physiol..

[79] Chu H.-H., Chiecko J., Punshon T., Lanzirotti A., Lahner B., Salt D.E., Walker E.L. (2010). Successful reproduction requires the function of Arabidopsis YELLOW STRIPE-LIKE1 and YELLOW STRIPE-LIKE3 metal-nicotianamine transporters in both vegetative and reproductive structures. Plant Physiol..

[80] Kim S.A., Punshon T., Lanzirotti A., Li L., Alonso J.M., Ecker J.R., Kaplan J., Guerinot M.L. (2006). Localization of iron in *Arabidopsis* seed requires the vacuolar membrane transporter VIT1. Science.

[81] Thomine S., Lelièvre F., Debarbieux E., Schroeder J.I., Barbier-Brygoo H. (2003). AtNRAMP3, a multispecific vacuolar metal transporter involved in plant responses to iron deficiency. Plant J..

[82] Segond D., Dellagi A., Lanquar V., Rigault M., Patrit O., Thomine S., Expert D. (2009). *NRAMP* genes function in *Arabidopsis thaliana* resistance to *Erwinia chrysanthemi* infection. Plant J..

[83] Bashir K., Takahashi R., Akhtar S., Ishimaru Y., Nakanishi H., Nishizawa N.K. (2013). The knockdown of *OsVIT2* and *MIT* affects iron localization in rice seed. Rice.

[84] Zhang Y., Xu Y.-H., Yi H.-Y., Gong J.-M. (2012). Vacuolar membrane transporters OsVIT1 and OsVIT2 modulate iron translocation between flag leaves and seeds in rice. Plant J..

[85] Nevo Y., Nelson N. (2006). The NRAMP family of metal-ion transporters. Biochim. Biophys. Acta Mol. Cell Res..

[86] Moore K.L., Zhao F.J., Gritsch C.S., Tosi P., Hawkesford M.J., McGrath S.P., Shewry P.R., Grovenor C.R.M. (2012). Localisation of iron in wheat grain using high resolution secondary ion mass spectrometry. J. Cereal Sci..

[87] Beasley J.T., Bonneau J.P., Sánchez-Palacios J.T., Moreno-Moyano L.T., Callahan D.L., Tako E., Glahn R.P., Lombi E., Johnson A.A.T. (2019). Metabolic engineering of bread wheat improves grain iron concentration and bioavailability. Plant Biotechnol. J..

[88] Raboy V. (2001). Seeds for a better future: ‘low phytate’ grains help to overcome malnutrition and reduce pollution. Trends Plant Sci..

[89] Brinch-Pedersen H., Madsen C.K., Dionisio G., Holm P.B. (2012). High Expression Cereal Phytase Gene. https://patents.google.com/patent/WO2012146597A1/en.

[90] Gao F., Robe K., Gaymard F., Izquierdo E., Dubos C. (2019). The transcriptional control of iron homeostasis in plants: A tale of *bHLH* transcription factors?. Front. Plant Sci..

[91] Yuan Y.X., Zhang J., Wang D.W., Ling H.Q. (2005). *AtbHLH29* of *Arabidopsis thaliana* is a functional ortholog of tomato *FER* involved in controlling iron acquisition in strategy I plants. Cell Res..

[92] Ling H.-Q., Bauer P., Bereczky Z., Keller B., Ganal M. (2002). The tomato fer gene encoding a bHLH protein controls iron-uptake responses in roots. Proc. Natl. Acad. Sci. USA.

[93] Colangelo E.P., Guerinot M. (2004). Lou The essential basic helix-loop-helix protein FIT1 is required for the iron deficiency response. Plant Cell.

[94] Wu H., Ling H.Q. (2019). FIT-binding proteins and their functions in the regulation of Fe homeostasis. Front. Plant Sci..

[95] Long T.A., Tsukagoshi H., Busch W., Lahner B., Salt D.E., Benfey P.N. (2010). The *bHLH* transcription factor POPEYE regulates response to iron deficiency in *Arabidopsis* roots. Plant Cell.

[96] Lockhart J. (2020). Personal trainer: bHLH121 functions upstream of a transcriptional network of heavy lifters involved in balancing iron levels. Plant Cell.

[97] Palmer C.M., Hindt M.N., Schmidt H., Clemens S., Guerinot M.L. (2013). *MYB10* and *MYB72* are required for growth under iron-limiting conditions. PLoS Genet..

[98] Zamioudis C., Hanson J., Pieterse C.M.J. (2014). β-glucosidase BGLU42 is a MYB72-dependent key regulator of rhizobacteria-induced systemic resistance and modulates iron deficiency responses in *Arabidopsis* roots. New Phytol..

[99] Stringlis I.A., Yu K., Feussner K., De Jonge R., Van Bentum S., Van Verk M.C., Berendsen R.L., Bakker P.A.H.M., Feussner I., Pieterse C.M.J. (2018). MYB72-dependent coumarin exudation shapes root microbiome assembly to promote plant health. Proc. Natl. Acad. Sci. USA.

[100] Yan J.Y., Li C.X., Sun L., Ren J.Y., Li G.X., Ding Z.J., Zheng S.J. (2016). A *WRKY* transcription factor regulates Fe translocation under Fe deficiency. Plant Physiol..

[101] Bournier M., Tissot N., Mari S., Boucherez J., Lacombe E., Briat J.F., Gaymard F. (2013). *Arabidopsis* ferritin 1 (*AtFer1*) gene regulation by the phosphate starvation response 1 (AtPHR1) transcription factor reveals a direct molecular link between iron and phosphate homeostasis. J. Biol. Chem..

[102] Selote D., Samira R., Matthiadis A., Gillikin J.W., Long T.A. (2015). Iron-binding E3 ligase mediates iron response in plants by targeting basic helix-loop-helix transcription factors. Plant Physiol..

[103] Rodríguez-Celma J., Connorton J.M., Kruse I., Green R.T., Franceschetti M., Chen Y.T., Cui Y., Ling H.Q., Yeh K.C., Balk J. (2019). Arabidopsis BRUTUS-LIKE E3 ligases negatively regulate iron uptake by targeting transcription factor FIT for recycling. Proc. Natl. Acad. Sci. USA.

[104] Tissot N., Robe K., Gao F., Grant-Grant S., Boucherez J., Bellegarde F., Maghiaoui A., Marcelin R., Izquierdo E., Benhamed M. (2019). Transcriptional integration of the responses to iron availability in Arabidopsis by the bHLH factor ILR3. New Phytol..

[105] Grillet L., Lan P., Li W., Mokkapati G., Schmidt W. (2018). IRON MAN is a ubiquitous family of peptides that control iron transport in plants. Nat. Plants.

[106] Kawakami Y., Bhullar N.K. (2018). Molecular processes in iron and zinc homeostasis and their modulation for biofortification in rice. J. Integr. Plant Biol..

[107] Ogo Y., Nakanishi Itai R., Nakanishi H., Kobayashi T., Takahashi M., Mori S., Nishizawa N.K. (2007). The rice bHLH protein OsIRO2 is an essential regulator of the genes involved in Fe uptake under Fe-deficient conditions. Plant J..

[108] Zheng L., Ying Y., Wang L., Wang F., Whelan J., Shou H. (2010). Identification of a novel iron regulated basic helix-loop-helix protein involved in Fe homeostasis in *Oryza sativa*. BMC Plant Biol..

[109] Kobayashi T., Ogo Y., Itai R.N., Nakanishi H., Takahashi M., Mori S., Nishizawa N.K. (2007). The transcription factor IDEF1 regulates the response to and tolerance of iron deficiency in plants. Proc. Natl. Acad. Sci. USA.

[110] Kobayashi T., Itai R.N., Aung M.S., Senoura T., Nakanishi H., Nishizawa N.K. (2012). The rice transcription factor IDEF1 directly binds to iron and other divalent metals for sensing cellular iron status. Plant J..

[111] Kobayashi T., Ogo Y., Aung M.S., Nozoye T., Itai R.N., Nakanishi H., Yamakawa T., Nishizawa N.K. (2010). The spatial expression and regulation of transcription factors IDEF1 and IDEF2. Ann. Bot..

[112] Ogo Y., Kobayashi T., Itai R.N., Nakanishi H., Kakei Y., Takahashi M., Toki S., Mori S., Nishizawa N.K. (2008). A novel NAC transcription factor, IDEF2, that recognizes the iron deficiency-responsive element 2 regulates the genes involved in iron homeostasis in plants. J. Biol. Chem..

[113] Kobayashi T., Nagasaka S., Senoura T., Itai R.N., Nakanishi H., Nishizawa N.K. (2013). Iron-binding haemerythrin RING ubiquitin ligases regulate plant iron responses and accumulation. Nat. Commun..

[114] Zhang H., Li Y., Yao X., Liang G., Yu D. (2017). Positive Regulator of Iron Homeostasis1, OsPRI1, facilitates iron homeostasis. Plant Physiol..

[115] Zhang H., Li Y., Pu M., Xu P., Liang G., Yu D. (2020). *Oryza sativa* Positive Regulator of Iron Deficiency Response 2 (OsPRI2) and OsPRI3 are involved in the maintenance of Fe homeostasis. Plant Cell Environ..

[116] Wang L., Ying Y., Narsai R., Ye L., Zheng L., Tian J., Whelan J., Shou H. (2013). Identification of OsbHLH133 as a regulator of iron distribution between roots and shoots in *Oryza sativa*. Plant Cell Environ..

[117] Liang G., Zhang H., Li Y., Pu M., Yang Y., Li C., Lu C., Xu P., Yu D. (2020). *Oryza sativa* Fer-Like Fe Deficiency-Induced Transcription Factor (OsFIT/OsbHLH156) interacts with OsIRO2 to regulate iron homeostasis. J. Integr. Plant Biol..

[118] Wang S., Li L., Ying Y., Wang J., Shao J.F., Yamaji N., Whelan J., Ma J.F., Shou H. (2020). A transcription factor OsbHLH156 regulates Strategy II iron acquisition through localising IRO2 to the nucleus in rice. New Phytol..

[119] Fan H., Zhang Z., Wang N., Cui Y., Sun H., Liu Y., Wu H., Zheng S., Bao S., Ling H.Q. (2014). SKB1/PRMT5-mediated histone H4R3 dimethylation of Ib subgroup *bHLH* genes negatively regulates iron homeostasis in *Arabidopsis thaliana*. Plant J..

[120] Assuncao A.G.L., Herrero E., Lin Y.F., Huettel B., Talukdar S., Smaczniak C., Immink R.G.H., van Eldik M., Fiers M., Schat H. (2010). *Arabidopsis thaliana* transcription factors bZIP19 and bZIP23 regulate the adaptation to zinc deficiency. Proc. Natl. Acad. Sci. USA.

[121] Lilay G.H., Castro P.H., Campilho A., Assunção A.G.L. (2019). The Arabidopsis bZIP19 and bZIP23 activity requires zinc deficiency-insight on regulation from complementation lines. Front. Plant Sci..

[122] Kobayashi T. (2019). Understanding the complexity of iron sensing and signaling cascades in plants. Plant Cell Physiol..

[123] Brumbarova T., Bauer P., Ivanov R. (2015). Molecular mechanisms governing *Arabidopsis* iron uptake. Trends Plant Sci..

[124] Chen W.W., Yang J.L., Qin C., Jin C.W., Mo J.H., Ye T., Zheng S.J. (2010). Nitric oxide acts downstream of auxin to trigger root ferric-chelate reductase activity in response to iron deficiency in Arabidopsis. Plant Physiol..

[125] Blum A., Brumbarova T., Bauer P., Ivanov R. (2014). Hormone influence on the spatial regulation of *IRT1* expression in iron-deficient *Arabidopsis thaliana* roots. Plant Signal. Behav..

[126] Tian Q., Zhang X., Yang A., Wang T., Zhang W.H. (2016). CIPK23 is involved in iron acquisition of *Arabidopsis* by affecting ferric chelate reductase activity. Plant Sci..

[127] Gratz R., Manishankar P., Ivanov R., Köster P., Mohr I., Trofimov K., Steinhorst L., Meiser J., Mai H.J., Drerup M. (2019). CIPK11-dependent phosphorylation modulates FIT activity to promote Arabidopsis iron acquisition in response to calcium signaling. Dev. Cell.

[128] Chen C.L., Cui Y., Cui M., Zhou W.J., Wu H.L., Ling H.Q. (2018). A FIT-binding protein is involved in modulating iron and zinc homeostasis in *Arabidopsis*. Plant Cell Environ..

[129] Yang Y., Ou B., Zhang J., Si W., Gu H., Qin G., Qu L.J. (2014). The Arabidopsis mediator subunit MED16 regulates iron homeostasis by associating with EIN3/EIL1 through subunit MED25. Plant J..

[130] Zhang Y., Wu H., Wang N., Fan H., Chen C., Cui Y., Liu H., Ling H.Q. (2014). Mediator subunit 16 functions in the regulation of iron uptake gene expression in Arabidopsis. New Phytol..

[131] Adamski N.M., Borrill P., Brinton J., Harrington S.A., Marchal C., Bentley A.R., Bovill W.D., Cattivelli L., Cockram J., Contreras-Moreira B. (2020). A roadmap for gene functional characterisation in crops with large genomes: Lessons from polyploid wheat. Elife.

[132] Hao Y., Velu G., Peña R.J., Singh S., Singh R.P. (2014). Genetic loci associated with high grain zinc concentration and pleiotropic effect on kernel weight in wheat (*Triticum aestivum* L.). Mol. Breed..

[133] Velu G., Singh R.P., Crespo-Herrera L., Juliana P., Dreisigacker S., Valluru R., Stangoulis J., Sohu V.S., Mavi G.S., Mishra V.K. (2018). Genetic dissection of grain zinc concentration in spring wheat for mainstreaming biofortification in CIMMYT wheat breeding. Sci. Rep..

[134] Qiao K., Wang F., Liang S., Wang H., Hu Z., Chai T. (2019). Improved Cd, Zn and Mn tolerance and reduced Cd accumulation in grains with wheat-based cell number regulator TaCNR2. Sci. Rep..

[135] Qiao K., Wang F., Liang S., Wang H., Hu Z., Chai T. (2019). New biofortification tool: Wheat TaCNR5 enhances zinc and manganese tolerance and increases zinc and manganese accumulation in rice grains. J. Agric. Food Chem..

[136] Uauy C., Brevis J.C., Dubcovsky J. (2006). The high grain protein content gene *Gpc-B1* accelerates senescence and has pleiotropic effects on protein content in wheat. J. Exp. Bot..

[137] Uauy C., Distelfeld A., Fahima T., Blechl A., Dubcovsky J. (2006). A *NAC* gene regulating senescence improves grain protein, zinc, and iron content in wheat. Science.

[138] Waters B.M., Uauy C., Dubcovsky J., Grusak M.A. (2009). Wheat (*Triticum aestivum*) NAM proteins regulate the translocation of iron, zinc, and nitrogen compounds from vegetative tissues to grain. J. Exp. Bot..

[139] Yang R., Juhasz A., Zhang Y., Chen X., Zhang Y., She M., Zhang J., Maddern R., Edwards I., Diepeveen D. (2018). Molecular characterisation of the *NAM-1* genes in bread wheat in Australia. Crop Pasture Sci..

[140] Chen X., Song G., Zhang S., Li Y., Gao J., Shahidul I., Ma W., Li G., Ji W. (2017). The allelic distribution and variation analysis of the *NAM-B1* gene in Chinese wheat cultivars. J. Integr. Agric..

[141] Distelfeld A., Cakmak I., Peleg Z., Ozturk L., Yazici A.M., Budak H., Saranga Y., Fahima T. (2007). Multiple QTL-effects of wheat *Gpc-B1* locus on grain protein and micronutrient concentrations. Physiol. Plant..

[142] Asplund L., Hagenblad J., Leino M.W. (2010). Re-evaluating the history of the wheat domestication gene *NAM-B1* using historical plant material. J. Archaeol. Sci..

[143] Hagenblad J., Asplund L., Balfourier F., Ravel C., Leino M.W. (2012). Strong presence of the high grain protein content allele of *NAM-B1* in Fennoscandian wheat. Theor. Appl. Genet..

[144] Velu G., Singh R.P., Cardenas M.E., Wu B., Guzman C., Ortiz-Monasterio I. (2017). Characterization of grain protein content gene (*Gpc-B1*) introgression lines and its potential use in breeding for enhanced grain zinc and iron concentration in spring wheat. Acta Physiol. Plant..

[145] Valluru R., Reynolds M.P., Salse J. (2014). Genetic and molecular bases of yield-associated traits: A translational biology approach between rice and wheat. Theor. Appl. Genet..

[146] Connorton J.M., Jones E.R., Rodríguez-Ramiro I., Fairweather-Tait S., Uauy C., Balk J. (2017). Wheat vacuolar iron transporter TaVIT2 transports Fe and Mn and is effective for biofortification. Plant Physiol..

[147] Bhati K.K., Aggarwal S., Sharma S., Mantri S., Singh S.P., Bhalla S., Kaur J., Tiwari S., Roy J.K., Tuli R. (2014). Differential expression of structural genes for the late phase of phytic acid biosynthesis in developing seeds of wheat (*Triticum aestivum* L.). Plant Sci..

[148] Bhati K.K., Alok A., Kumar A., Kaur J., Tiwari S., Pandey A.K. (2016). Silencing of *ABCC13* transporter in wheat reveals its involvement in grain development, phytic acid accumulation and lateral root formation. J. Exp. Bot..

[149] Aggarwal S., Kumar A., Bhati K.K., Kaur G., Shukla V., Tiwari S., Pandey A.K. (2018). RNAi-mediated downregulation of inositol pentakisphosphate kinase (*IPK1*) in wheat grains decreases phytic acid levels and increases Fe and Zn accumulation. Front. Plant Sci..

[150] Peng J., Richards D.E., Hartley N.M., Murphy G.P., Devos K.M., Flintham J.E., Beales J., Fish L.J., Worland A.J., Pelica F. (1999). ‘Green Revolution’ genes encode mutant gibberellin response modulators. Nature.

[151] Zhang J., Dell B., Biddulph B., Drake-Brockman F., Walker E., Khan N., Wong D., Hayden M., Appels R. (2013). Wild-type alleles of *Rht-B1* and *Rht-D1* as independent determinants of thousand-grain weight and kernel number per spike in wheat. Mol. Breed..

[152] DeFries R., Fanzo J., Remans R., Palm C., Wood S., Anderman T.L. (2015). Metrics for land-scarce agriculture. Science.

[153] Velu G., Singh R.P., Huerta J., Guzmán C. (2017). Genetic impact of *Rht* dwarfing genes on grain micronutrients concentration in wheat. F. Crop. Res..

[154] Jobson E.M., Martin J.M., Schneider T.M., Giroux M.J. (2018). The impact of the *Rht-B1b*, *Rht-D1b*, and *Rht-8* wheat semi-dwarfing genes on flour milling, baking, and micronutrients. Cereal Chem..

[155] Yasmin Z. (2013). Investigating the Genetics and Agronomic Traits Associated with Elevated Grain Zn Concentration in Wheat. Ph.D. Thesis.

[156] Rasheed A., Xia X. (2019). From markers to genome-based breeding in wheat. Theor. Appl. Genet..

[157] Ozkan H., Brandolini A., Torun A., AltIntas S., Eker S., Kilian B., Braun H.J., Salamini F., Cakmak I., Buck H.T., Nisi J.E., Salomon N. (2007). Natural variation and identification of microelements content in seeds of einkorn wheat (*Triticum monococcum*). Wheat Production in Stressed Environments.

[158] Shi R., Li H., Tong Y., Jing R., Zhang F., Zou C. (2008). Identification of quantitative trait locus of zinc and phosphorus density in wheat (*Triticum aestivum* L.) grain. Plant Soil.

[159] Genc Y., Verbyla A.P., Torun A.A., Cakmak I., Willsmore K., Wallwork H., McDonald G.K. (2009). Quantitative trait loci analysis of zinc efficiency and grain zinc concentration in wheat using whole genome average interval mapping. Plant Soil.

[160] Peleg Z., Cakmak I., Ozturk L., Yazici A., Jun Y., Budak H., Korol A.B., Fahima T., Saranga Y. (2009). Quantitative trait loci conferring grain mineral nutrient concentrations in durum wheat × wild emmer wheat RIL population. Theor. Appl. Genet..

[161] Tiwari V.K., Rawat N., Chhuneja P., Neelam K., Aggarwal R., Randhawa G.S., Dhaliwal H.S., Keller B., Singh K. (2009). Mapping of quantitative trait loci for grain iron and zinc concentration in diploid a genome wheat. J. Hered..

[162] Tiwari C., Wallwork H., Arun B., Mishra V.K., Velu G., Stangoulis J., Kumar U., Joshi A.K. (2016). Molecular mapping of quantitative trait loci for zinc, iron and protein content in the grains of hexaploid wheat. Euphytica.

[163] Roshanzamir H., Kordenaeej A., Bostani A. (2013). Mapping QTLs related to Zn and Fe concentrations in bread wheat (*Triticum aestivum*) grain using microsatellite markers. Iran. J. Genet. Plant Breed..

[164] Shi R., Tong Y., Jing R., Zhang F., Zou C. (2013). Characterization of quantitative trait loci for grain minerals in hexaploid wheat (*Triticum aestivum* L.). J. Integr. Agric..

[165] Pu Z.E., Yu M., He Q.Y., Chen G.Y., Wang J.R., Liu Y.X., Jiang Q.T., Li W., Dai S.F., Wei Y.M. (2014). Quantitative trait loci associated with micronutrient concentrations in two recombinant inbred wheat lines. J. Integr. Agric..

[166] Srinivasa J., Arun B., Mishra V.K., Singh G.P., Velu G., Babu R., Vasistha N.K., Joshi A.K. (2014). Zinc and iron concentration QTL mapped in a *Triticum spelta* × *T. aestivum* cross. Theor. Appl. Genet..

[167] Yasmin Z., Paltridge N., Graham R., Huynh B.L., Stangoulis J. (2014). Measuring genotypic variation in wheat seed iron first requires stringent protocols to minimize soil iron contamination. Crop Sci..

[168] Xu Y., An D., Liu D., Zhang A., Xu H., Li B. (2012). Molecular mapping of QTLs for grain zinc, iron and protein concentration of wheat across two environments. F. Crop. Res..

[169] Crespo-Herrera L.A., Velu G., Singh R.P. (2016). Quantitative trait loci mapping reveals pleiotropic effect for grain iron and zinc concentrations in wheat. Ann. Appl. Biol..

[170] Crespo-Herrera L.A., Govindan V., Stangoulis J., Hao Y., Singh R.P. (2017). QTL mapping of grain Zn and Fe concentrations in two hexaploid wheat RIL populations with ample transgressive segregation. Front. Plant Sci..

[171] Krishnappa G., Singh A.M., Chaudhary S., Ahlawat A.K., Singh S.K., Shukla R.B., Jaiswal J.P., Singh G.P., Solanki I.S. (2017). Molecular mapping of the grain iron and zinc concentration, protein content and thousand kernel weight in wheat (*Triticum aestivum* L.). PLoS ONE.

[172] Velu G., Tutus Y., Gomez-Becerra H.F., Hao Y., Demir L., Kara R., Crespo-Herrera L.A., Orhan S., Yazici A., Singh R.P. (2017). QTL mapping for grain zinc and iron concentrations and zinc efficiency in a tetraploid and hexaploid wheat mapping populations. Plant Soil.

[173] Liu J., Wu B., Singh R.P., Velu G. (2019). QTL mapping for micronutrients concentration and yield component traits in a hexaploid wheat mapping population. J. Cereal Sci..

[174] Guttieri M.J., Stephen Baenziger P., Frels K., Carver B., Arnall B., Wang S., Akhunov E., Waters B.M. (2015). Prospects for selecting wheat with increased zinc and decreased cadmium concentration in grain. Crop Sci..

[175] Manickavelu A., Hattori T., Yamaoka S., Yoshimura K., Kondou Y., Onogi A., Matsui M., Iwata H., Ban T. (2017). Genetic nature of elemental contents in wheat grains and its genomic prediction: Toward the effective use of wheat landraces from Afghanistan. PLoS ONE.

[176] Gorafi Y.S.A., Ishii T., Kim J.S., Elbashir A.A.E., Tsujimoto H. (2018). Genetic variation and association mapping of grain iron and zinc contents in synthetic hexaploid wheat germplasm. Plant Genet. Resour. Charact. Util..

[177] Alomari D.Z., Eggert K., von Wirén N., Alqudah A.M., Polley A., Plieske J., Ganal M.W., Pillen K., Röder M.S. (2018). Identifying candidate genes for enhancing grain Zn concentration in wheat. Front. Plant Sci..

[178] Alomari D.Z., Eggert K., Von Wirén N., Polley A., Plieske J., Ganal M.W., Liu F., Pillen K., Röder M.S. (2019). Whole-genome association mapping and genomic prediction for iron concentration in wheat grains. Int. J. Mol. Sci..

[179] Kumar J., Saripalli G., Gahlaut V., Goel N., Meher P.K., Mishra K.K., Mishra P.C., Sehgal D., Vikram P., Sansaloni C. (2018). Genetics of Fe, Zn, β-carotene, GPC and yield traits in bread wheat (*Triticum aestivum* L.) using multi-locus and multi-traits GWAS. Euphytica.

[180] Jackson P., Robertson M., Cooper M., Hammer G. (1996). The role of physiological understanding in plant breeding: From a breeding perspective. F. Crop. Res..

[181] Xia H.Y., Wang L., Qiao Y.T., Kong W.L., Xue Y.H., Wang Z.S., Kong L.G., Xue Y.F., Sizmur T. (2020). Elucidating the source–sink relationships of zinc biofortification in wheat grains: A review. Food Energy Secur..

[182] Chen X.P., Zhang Y.Q., Tong Y.P., Xue Y.F., Liu D.Y., Zhang W., Deng Y., Meng Q.F., Yue S.C., Yan P. (2017). Harvesting more grain zinc of wheat for human health. Sci. Rep..

[183] Cao S., Xu D., Hanif M., Xia X., He Z. (2020). Genetic architecture underpinning yield component traits in wheat. Theor. Appl. Genet..

[184] Gupta P.K., Balyan H.S., Sharma S., Kumar R. (2020). Genetics of yield, abiotic stress tolerance and biofortification in wheat (*Triticum aestivum* L.). Theor. Appl. Genet..

[185] Henry R.J., Furtado A., Rangan P. (2018). Wheat seed transcriptome reveals genes controlling key traits for human preference and crop adaptation. Curr. Opin. Plant Biol..

[186] Hefferon K. (2019). Biotechnological approaches for generating zinc-enriched crops to combat malnutrition. Nutrients.

[187] Connorton J.M., Balk J. (2019). Iron biofortification of staple crops: Lessons and challenges in plant genetics. Plant Cell Physiol..

[188] Sui X., Zhao Y., Wang S., Duan X., Xu L., Liang R. (2012). Improvement Fe content of wheat (*Triticum aestivum*) grain by soybean ferritin expression cassette without vector backbone sequence. J. Agric. Biotechnol..

[189] Borg S., Brinch-Pedersen H., Tauris B., Madsen L.H., Darbani B., Noeparvar S., Holm P.B. (2012). Wheat ferritins: Improving the iron content of the wheat grain. J. Cereal Sci..

[190] Singh S.P., Keller B., Gruissem W., Bhullar N.K. (2017). Rice *NICOTIANAMINE SYNTHASE 2* expression improves dietary iron and zinc levels in wheat. Theor. Appl. Genet..

[191] Petolino J.F., Srivastava V., Daniell H. (2016). Editing plant genomes: A new era of crop improvement. Plant Biotechnol. J..

[192] Hao Y., Rasheed A., Jackson R., Xiao Y., Zhang Y., Xia X., He Z. (2020). Advanced genomics and breeding tools to accelerate the development of climate resilient wheat. Genomic Designing of Climate-Smart Cereal Crops.

[193] Wang K., Gong Q., Ye X. (2020). Recent developments and applications of genetic transformation and genome editing technologies in wheat. Theor. Appl. Genet..

[194] Li J., Li H., Chen J., Yan L., Xia L. (2020). Toward precision genome editing in crop plants. Mol. Plant.

[195] Lin Q., Zong Y., Xue C., Wang S., Jin S., Zhu Z., Wang Y., Anzalone A.V., Raguram A., Doman J.L. (2020). Prime genome editing in rice and wheat. Nat. Biotechnol..

[196] Callaway E. (2018). CRISPR plants now subject to tough GM laws in European Union. Nature.

[197] Tabbita F., Pearce S., Barneix A.J. (2017). Breeding for increased grain protein and micronutrient content in wheat: Ten years of the *Gpc-B1* gene. J. Cereal Sci..

[198] Xu Y., Li P., Zou C., Lu Y., Xie C., Zhang X., Prasanna B.M., Olsen M.S. (2017). Enhancing genetic gain in the era of molecular breeding. J. Exp. Bot..

[199] Heffner E.L., Lorenz A.J., Jannink J.L., Sorrells M.E. (2010). Plant breeding with genomic selection: Gain per unit time and cost. Crop Sci..

[200] Li L., Fang Z., Zhou J., Chen H., Hu Z., Gao L., Chen L., Ren S., Ma H., Lu L. (2017). An accurate and efficient method for large-scale SSR genotyping and applications. Nucleic Acids Res..

[201] Guo Z., Wang H., Tao J., Ren Y., Xu C., Wu K., Zou C., Zhang J., Xu Y. (2019). Development of multiple SNP marker panels affordable to breeders through genotyping by target sequencing (GBTS) in maize. Mol. Breed..

[202] Liu H.J., Yan J. (2019). Crop genome-wide association study: A harvest of biological relevance. Plant J..

[203] Wang M., Yuan J., Qin L., Shi W., Xia G., Liu S. (2020). *TaCYP81D5*, one member in a wheat cytochrome P450 gene cluster, confers salinity tolerance via reactive oxygen species scavenging. Plant Biotechnol. J..

